# Time to say goodbye

**DOI:** 10.1590/1516-3180.2014.9431326

**Published:** 2014-09-02

**Authors:** Paulo Manuel Pêgo-Fernandes

**Affiliations:** I MD, PhD. Titular Professor, Discipline of Thoracic Surgery, Instituto do Coração (InCor), Hospital das Clínicas (HC), Faculdade de Medicina da Universidade de São Paulo (FMUSP), São Paulo, Brazil

Dear reader,

Since 2009, when Prof. Dr. Álvaro Nagib Atallah and I accepted the challenge of leading the Scientific Department of Associação Paulista de Medicina (APM), I have had the privilege of also taking on the function of editor of the journals of this entity: São Paulo Medical Journal and Diagnóstico & Tratamento.

From the outset, this was seen to be an arduous task that would certainly be greater than any prediction that we might have made. We discovered that fulfilling this task with the deserved quality was only saved from being impossible thanks to the support and collaboration of a multiprofessional team that dedicated itself to the APM every day. This team became ever more professional as time went by, with the aim of providing our association with the best resources available in all areas. 

After completing six years of leading this undertaking (three years as Adjunct Director and three years as Director), the time has come to "pass the baton", by handing over this position that I have served with pride and dedication. Nonetheless, this is not just a moment of farewell but, rather, a moment of renewal, and for this reason I would like to reflect on what has been achieved.

Specifically with regard to the São Paulo Medical Journal, the most important objective was to raise the relevance of the journal within the international scenario, without losing its focus on dissemination of general medical knowledge. In other words, we sought articles for the journal that would have high quality and scientific rigor, but without transforming the journal into something very specialized or that might cause habitual readers of the journal to drift away. The crowning moment of this work was certainly when, for the first time, the São Paulo Medical Journal obtained a designated impact factor from Thompson Reuters.

In relation to Diagnóstico & Tratamento, we maintained the premise of disseminating medical knowledge in a wide-ranging manner through sections dedicated to specialties (gynecology, dermatology, nutrology etc.) and the sections Patient-Oriented Evidence that Matters (POEMs), Evidence-Based Medicine, Language, Residency and Medical Teaching, General Interest, Opinion and Cochrane Highlights.

At the helm of the Scientific Department, where I initially held the position of Adjunct Scientific Director until 2012 and then Scientific Director until the present date, I sought to encourage initiatives that would bring greater dynamism and penetration for the APM. One successful example of this is the Student Medical Congress, which was first held in 2009 and translated very well our intention to interact with physicians from the time of their graduation onwards, by stimulating them and assisting them in their technical and scientific development.

Other activities of the Scientific Department that deserve highlighting are the interaction and partnerships with health departments and universities that have had the aim of developing medical teaching and scientific updating. Building ties with specialty societies was a partially achieved objective, and this will certainly end up being consolidated through the work of the next board of directors. 

I would like to conclude by thanking all of those involved in the journal's editorial process, the entire board of directors of the Associação Paulista de Medicina and, especially, our readers, who are the major motive for our work.

